# Publications of Mass. Homœopathic Medical Society

**Published:** 1887-09

**Authors:** 


PUBLICATIONS OF THE MASSACHUSETTS HOMŒOPATHIC MEDICAL SOCIETY. Volume IX. 1886.

This work is a volume of transactions including many valuable papers on Ozone, Hysteria, "Calc. phos. and Podophyl. in Entero-Colitis," "Atmospheric Humidity in Relation to Disease," etc.

